# Agro-Industrial Wastes for Production of Biosurfactant by *Bacillus subtilis* ANR 88 and Its Application in Synthesis of Silver and Gold Nanoparticles

**DOI:** 10.3389/fmicb.2017.00492

**Published:** 2017-03-24

**Authors:** Ashwini N. Rane, Vishakha V. Baikar, V. Ravi Kumar, Rajendra L. Deopurkar

**Affiliations:** ^1^Department of Microbiology, Savitribai Phule Pune UniversityPune, India; ^2^Department of Environmental Science, Savitribai Phule Pune UniversityPune, India; ^3^Chemical Engineering and Process Development Division, CSIR-National Chemical LaboratoryPune, India

**Keywords:** biosurfactant, agro-industrial waste, nanoparticles, *Bacillus subtilis*, Plackett-Burman design-optimization

## Abstract

Biosurfactants, surface-active amphiphilic compounds, despite having a wide range of applications, have a high cost of production, which severely restricts their use. For cheaper production of biosurfactant, we investigated the potential of the indigenously isolated biosurfactant producing organism, *Bacillus subtilis* ANR 88, to grow on different cheap carbon sources (molasses, whey, and extracts of potato peels, orange peels, banana peels, and bagasse). We found that, *B. subtilis* ANR 88 used significant amounts of total sugar to produce cell biomass and biosurfactant. The biosurfactant production in minimal medium containing glucose as sole source of carbon was 0.207 g/l and the same with molasses as carbon source was 0.241 g/l. With whey as carbon source, isolate failed to produce biosurfactant. Amongst the extracts of the agro-wastes, the extracts of bagasse and orange peels gave 0.127 and 0.089 g/l of biosurfactant respectively. One-variable-at-a-time (OVAT) studies carried out to optimize the production of biosurfactant by *B. subtilis* ANR 88 resulted into maximum biosurfactant yield of 0.513 g/l in medium: molasses 4%, ammonium ferric citrate 0.25%, pH 7. Plackett–Burman design based statistical method for optimization increased the production of biosurfactant to 0.746 g/l, which is 3.6-fold of that produced on glucose. The biosurfactant produced by *B. subtilis* ANR 88 was analyzed by Fourier Transform Infrared Spectroscopy (FT-IR); it showed that the biosurfactant contained alkyl as well as peptide groups. The biosurfactant of *B. subtilis* ANR 88 was found effective in the synthesis of silver as well as gold nanoparticles in the total absence of conventional chemical reducing agents. Interestingly, nanoparticles produced were almost uniform in their size and shapes i.e., spherical silver (4–18 nm) and hexagonal gold nanoparticles (40–60 nm), as evident in TEM images.

## Introduction

Biosurfactants are surface-active amphiphilic compounds produced by variety of microorganisms, primarily to increase the bioavailability, solubility, and biodegradation of hydrophobic organic compounds present in their environment. Desai and Banat (1997) extensively reviewed the biosurfactants with respect to their chemistry, physiology, genetics, fermentative production and potential commercial applications. Further, Cameotra and Makkar (2004) and Rodrigues et al. (2006) highlighted the immunological and medical applications of the biosurfactants. Despite their vast array of applications, high cost of production severely restricts the use of biosurfactants. Optimization of growth and production parameters and use of cheap raw materials like agro-wastes, are thought to enhance the cost effectiveness of the biosurfactant production process (Nitschke et al., 2004; Benincasa, 2007; Joshi et al., 2008a).

Ability of the biosurfactants to act both as reducing as well as stabilizing agents in the synthesis of nanoparticles have recently been reported by Priyadarshini et al. (2016). Nanoparticles are usually synthesized by physicochemical methods that involve the use of high pressure, temperature and energy; as suggested by Plaza et al. (2014) the synthesis of nanoparticles using chemical methods are usually associated with the formation of toxic by-products. Therefore, the use of biosurfactants/biological materials for synthesis and stabilization of nanoparticles is gaining importance since the last decade (Kasture et al., 2008; Kumar C. G. et al., 2010).

The aim of the present work was to analyse the use of cheap agro-industrial wastes, *viz*., molasses, whey, extracts of potato peels, orange peels, banana peels, and bagasse for the production of biosurfactant using indigenously isolated *Bacillus subtilis* ANR 88. A further aim was to explore Plackett-Burman statistical approach to enhance the yield of biosurfactant. We also report here the synthesis of nanoparticles using the biosurfactant from *B. subtilis* ANR 88 grown on non-hydrocarbon agro-industrial wastes.

## Materials and methods

### Organism and culture conditions

*Bacillus subtilis* ANR 88 (GenBank accession number: KX670429) was isolated from local canteen waste and it was stored as frozen stock cultures at −80°C in 35% glycerol.

### Inoculum and media preparation

The inoculum was prepared in nutrient broth incubated at 30°C for 24 h. The minimal medium containing (g/L), ammonium nitrate, 1.0; potassium di-hydrogen phosphate, 6.0; di-hydrogen phosphate, 2.7; magnesium sulfate heptahydrate, 0.1 and trace elements (mg/L) calcium chloride, 1.2; ferrous sulfate heptahydrate, 1.65; manganese sulfate tetrahydrate,1.5; sodium salt of EDTA, 2.2 was used as basal medium. Different carbon sources were added separately in the autoclaved medium. Aqueous extracts of the agro-wastes *viz*., orange peels, potato peels, banana peels, and bagasse were prepared using the procedure described by Kulkarni et al. (2015). Stock solutions of agro-wastes were prepared by autoclaving 10% (w/v) suspensions of each dried waste. The extracts were filtered through muslin cloth and Whatman filter paper no.1 and clear filtrates were re-autoclaved. These extracts were added in the medium at 4% (v/v). Desired quantities of molasses were separately autoclaved in distilled water and added to the medium.

### Biosurfactant extraction and purification

In order to produce biosurfactant, the cultures were grown at 30°C for 24 h, in media prepared as described above. The crude biosurfactant was obtained by centrifuging the culture at 12,000 rpm for 30 min at 4°C. The surface tension measurements of the culture supernatants were carried out using a DCAT 11 Tensiometer (DataPhysics Corporation, Germany) equipped with Wilhelmy plate. The biosurfactant was purified by acid precipitation. The supernatant was acidified with 6 N HCl and kept for precipitation at 4°C overnight. The precipitate was collected by centrifugation at 12,000 rpm for 30 min at 4°C and was then suspended in minimum volume of distilled water, neutralized with 1 N NaOH and kept on magnetic stirrer for 1 h to dissolve it completely. The biosurfactant was extracted from the aqueous solution according to the method described by Ghribi et al. (2012), wherein the aqueous solution was mixed with equal volume of chloroform: methanol (2:1) and organic phase was collected. The procedure was repeated at least thrice to ensure maximum biosurfactant recovery.

### Effect of carbon source on growth and biosurfactant production

Different agro-industrial wastes were studied for their potential to act as a sole source of carbon for the biosurfactant production. Aqueous extracts of the agro-wastes 4% (v/v) were used for the biosurfactant production keeping all the other salts at their basal levels as mentioned earlier.

### One-variable at a-time (OVAT) studies for growth and biosurfactant production

The serial optimization approach was followed by varying the concentration of one-variable-at-a-time (OVAT) while keeping all others at their chosen levels. Once a parameter was optimized, this optimum level was used for the next round of OVAT studies conducted with the other variable and so on.

First, the effect of different nitrogen sources (ammonium nitrate, ammonium ferric citrate, ammonium sulfate, ammonium chloride, potassium nitrate, sodium nitrate, urea, yeast extract, peptone) on biomass, and biosurfactant production was determined. An uninoculated control flask was also maintained. The flasks were maintained at 30°C in an incubator shaker at 160 rpm.

Subsequently, the effects of various environmental factors, such as, pH (5–8), inoculum size (1–5%) and incubation temperature (20–37°C) on the growth and biosurfactant production by *B. subtilis* ANR 88 were determined.

### Time course of biosurfactant production in optimal medium

The effect of incubation time on the growth and biosurfactant production in OVAT optimized medium was monitored over the period of 72 h. Growth was measured in terms of biomass (g/l) while biosurfactant was extracted, purified, and expressed in terms of its dry weight.

### Determination of critical media components for biosurfactant production

A statistical set of experiments based on the Plackett-Burman design was carried out to determine the significance of the various medium constituents in the production of biosurfactant by *B. subtilis* ANR 88. PBD assumes that there are no or negligible interactions between the variables, i.e., critical media constituents, under consideration and a linear model with no interactive terms followed, *viz*.

(1) $$\begin{array}{r} Y=\beta_{0}+\sum \beta_{i}x_{i}\;\left(i=1,\ldots \ldots k\right)\ldots \end{array}$$

where, *x*_*i*_ and β_*i*_ are the regression coefficients and *Y* is the estimated response (Mukherjee et al., 2008).

In the present work, effect of 11 factors at two levels (+1 and –1) in terms of biosurfactant yield by the isolate *B. subtilis* ANR 88 was determined in 12 experiments as per the PBD (Table 1). Factors taken into consideration included media components, *viz*., molasses, ammonium ferric citrate, magnesium sulfate, ferrous sulfate, calcium chloride, manganese sulfate, di-sodium phosphate, potassium di-hydrogen phosphate, and environmental factors, such as pH, inoculum size, and incubation time. Design Expert Ver. 7.0.2., (Stat Ease Inc., Minneapolis, USA) was used for obtaining the statistical design for these experiments.

**Table 1:** **Various media components studied in PBD experiments and their chosen high and low concentration levels**.

**Variables code**	**Media constituents**	**Low level (−)**	**High level (+)**
A	(NH_4_)_5_Fe(C_6_H_4_O_7_)_2_ (g/l)	1	3
B	KH_2_PO_4_ (g/l)	5	7
C	Na_2_HPO_4_ (g/l)	2	3
D	MgSO_4_.7H_2_O (g/l)	0.05	1.5
E	CaCl_2_ (mg/l)	1	1.4
F	FeSO_4_.7H_2_O (mg/l)	1.5	1.8
G	MnSO_4_.4H_2_O (mg/l)	1	2
H	pH	6	8
J	Inoculum size (%)	1	5
K	Incubation Period (h)	36	60
L	Molasses (%)	3	5

Media were prepared using the 12 combinations of factors and inoculated with the stated amount of overnight grown inoculum. Flasks were incubated on the shaker at 160 rpm, at 30°C for different time periods. All the experiments were performed in triplicate and average of the three responses are reported. The linear model was obtained and ANOVA analysis was carried out for validation of the model.

### Characterization of biosurfactant by fourier transform infrared spectroscopy (FT-IR)

The biosurfactant was mixed with KBr (Spectroscopic Grade) and the infrared spectra (with wave numbers ranging from 4,000 to 400 cm^−1^) were recorded in Shimadzu FT-IR-8400 spectrometer. The data collected were the average of 50 scans over the entire range.

### Production of silver and gold nanoparticles from biosurfactant

The biosurfactant from the *B. subtilis* ANR 88 was extracted and purified as described earlier. The biosurfactant dried out from chloroform:methanol in the procedure was used for the synthesis of silver and gold nanoparticles (SNPs and GNPs). The mixture containing AgNO_3_ (for silver nanoparticles synthesis) or HAuCl_4_ (for gold nanoparticles synthesis) and biosurfactant were incubated at 90°C in micro-titer plate (96-well) for appropriate time period leading to the formation of nanoparticles. Nanoparticles syntheses were optimized in terms of AgNO_3_ or HAuCl_4_ and biosurfactant concentration. All the above experiments were monitored on UV-Vis spectrophotometer (Spectramax M2e) at an interval of 10 nm (200–800 nm) for 2 h. The effect of temperature was studied in the range of 40–90°C.

## Results

### Effect of carbon source on growth and biosurfactant production

Use of cheap raw materials for the growth and production is a common strategy, for reducing the production cost of biosurfactants. Whey, molasses, and aqueous extracts of agro-industrial wastes like potato peels, banana peels, orange peels, and sugar cane bagasse were used in the production media; minimal medium containing glucose was used as control. Specific yields were calculated as the ratio of biomass to utilized sugar and biosurfactant to utilized sugar.

As can be seen from Table 2, *B. subtilis* ANR 88 grew on all the substrates used in the study. The isolate produced same amount of biosurfactant when grown on molasses (0.241 g/l) as that on glucose (0.207 g/l); the yields with extracts of banana peels, orange peels and bagasse were 0.049, 0.089, and 0.127 g/l. There was no biosurfactant produced with whey as carbon source. Biosurfactant production on all the substrates compared on the basis of sugar utilized; molasses and glucose gave 0.017 g of biosurfactant per g of sugar utilized, while, the potato peels extract gave 0.032 g of biosurfactant per g of sugar utilized.

**Table 2:** **Biomass and biosurfactant yield from ***B. subtilis*** ANR 88 grown on agro-industrial residues**.

**Substrate**	**Initial sugar (g/l)**	**Utilized sugar (g/l)**	**Biomass (g/l)**	**Biomass/utilized sugar (g/g)**	**Biosurfactant yield (g/l)**	**Biosurfactant yield/utilized sugar (g/g)**
Glu	23.515 ± 0.919	11.214 ± 0.441	1.272 ± 0.034	0.103	0.207 ± 0.001	0.017
Mol	27.928 ± 1.150	13.416 ± 1.735	3.531 ± 0.190	0.243	0.241 ± 0.006	0.017
Whey	14.857 ± 0.401	8.137 ± 0.100	0.179 ± 0.023	0.027	0.000 ± 0.000	0.000
OgE	18.551 ± 0.776	0.481 ± 0.070	0.315 ± 0.029	0.017	0.089 ± 0.001	0.005
PPE	0.798 ± 0.260	0.134 ± 0.003	0.313 ± 0.001	0.470	0.022 ± 0.001	0.032
BgE	12.871 ± 0.891	0.198 ± 0.009	0.163 ± 0.004	0.013	0.127 ± 0.002	0.010
BnE	8.322 ± 1.659	0.264 ± 0.039	0.152 ± 0.004	0.019	0.049 ± 0.000	0.006

*Glu, Glucose; PPE, Potato peels extract; BgE, Bagasse extract; BnE, Banana peels extract; OgE, Orange peels extract; Mol, Molasses*.

*Results are expressed as the average ± SD of three independent measurements*.

### OVAT studies on growth and biosurfactant production by *B. subtilis* ANR 88

The OVAT optimization studies were carried out as an indicator to decide on the levels of the media constituents to be used for PBD. Table 3 shows the effects of molasses concentration, nitrogen source, and its concentration, temperature, pH and inoculum size on the growth and biosurfactant production by *B. subtilis* ANR 88 using the OVAT approach. It was observed that biomass increased till 10% molasses concentration although the biosurfactant yield did not increase significantly after 4% molasses concentration. Therefore, the effect of different nitrogen sources was investigated using 4% molasses as the carbon source; it was observed that biosurfactant production was in the range of 0.1–0.247 g/l and it did not vary considerably with the nature of the nitrogen source (i.e., organic/inorganic). However, the growth of the isolate was better with organic nitrogen sources, *viz*., urea, yeast extract and peptone.

**Table 3:** **Effect of various nutrients and environmental conditions on the growth and biosurfactant production by ***B. subtilis*** ANR 88**.

**Parameters**	**Biosurfactant yield g/l**	**Biomass g/l**
**MOLASSES (%)**		
1.0	0.090 ± 0.021	0.334 ± 0.001
2.0	0.141 ± 0.023	0.964 ± 0.002
3.0	0.125 ± 0.210	0.987 ± 0.003
**4.0**	**0.190 ± 0.005**	**2.904 ± 0.000**
5.0	0.179 ± 0.043	3.148 ± 0.006
6.0	0.190 ± 0.004	3.778 ± 0.011
7.0	0.151 ± 0.001	5.108 ± 0.009
8.0	0.166 ± 0.008	5.661 ± 0.012
9.0	0.183 ± 0.002	6.010 ± 0.011
10.0	0.189 ± 0.009	6.853 ± 0.090
**NITROGEN SOURCES**		
Ammonium Nitrate	0.190 ± 0.005	2.904 ± 0.000
**Ammonium ferric citrate**	**0.229 ± 0.020**	**4.196 ± 0.090**
Ammonium sulfate	0.115 ± 0.026	3.431 ± 0.121
Ammonium chloride	0.100 ± 0.010	3.590 ± 0.091
Potassium nitrate	0.253 ± 0.126	3.624 ± 0.188
Sodium nitrate	0.240 ± 0.170	2.904 ± 0.002
Urea	0.203 ± 0.000	3.664 ± 0.073
Yeast extract	0.193 ± 0.008	3.415 ± 0.349
Peptone	0.247 ± 0.017	3.562 ± 0.002
**AMMONIUM FERRIC CITRATE (%)**		
0.05	0.240 ± 0.018	3.873 ± 0.001
0.1	0.267 ± 0.008	3.609 ± 0.001
0.15	0.313 ± 0.009	4.866 ± 0.020
0.2	0.310 ± 0.023	4.626 ± 0.006
**0.25**	**0.360 ± 0.011**	**4.842 ± 0.002**
0.3	0.346 ± 0.019	5.202 ± 0.015
0.35	0.353 ± 0.013	5.596 ± 0.004
**INCUBATION TEMPERATURE** °**C**		
20	0.056 ± 0.013	4.656 ± 0.202
25	0.175 ± 0.024	5.076 ± 0.069
**30**	**0.372 ± 0.033**	**5.789 ± 0.105**
37	0.345 ± 0.028	5.900 ± 0.067
**pH**		
5.0	0.122 ± 0.014	4.353 ± 0.186
6.0	0.355 ± 0.008	4.972 ± 0.086
**7.0**	**0.457 ± 0.018**	**5.839 ± 0.008**
8.0	0.440 ± 0.013	5.409 ± 0.026
**INOCULUM SIZE (%)**		
1.0	0.481 ± 0.01	6.296 ± 0.331
**2.0**	**0.513 ± 0.01**	**4.929 ± 0.327**
3.0	0.464 ± 0.02	5.865 ± 0.071
4.0	0.469 ± 0.02	5.743 ± 0.103
5.0	0.475 ± 0.06	5.787 ± 0.077

*Results are expressed as the average ± SD of three independent measurements*.

*The optimal parameters and values are shown in bold*.

It is clear from Table 3 that there was a steady increase in biosurfactant yield with increase in concentration of ammonium ferric citrate up to 0.25%. The biosurfactant yield obtained at 0.25% ammonium ferric citrate was 0.360 g/l, which is one-and-half fold more than that obtained (0.240 g/l) at 0.05% concentration of ammonium ferric citrate. Biomass yields in the range of concentrations of ammonium ferric citrate studied lay in the range of (3.873–5.596 g/l). Acidic pH showed inhibitory effect on the biosurfactant and biomass production, by *B. subtilis* ANR 88. The isolate produced 0.122 g/l of biosurfactant at pH 5, and 0.457 g/l and 0.440 g/l at pH 7 and 8 respectively. Data showing the effect of incubation temperature indicated that 30°C is the optimum temperature for maximum biosurfactant production (0.372 g/l). As shown in Table 3, the maximum biosurfactant yield of 0.513 g/l was obtained on employing 2% concentration of the inoculum.

### Effect of incubation period on growth and biosurfactant production

**Figure 1:**
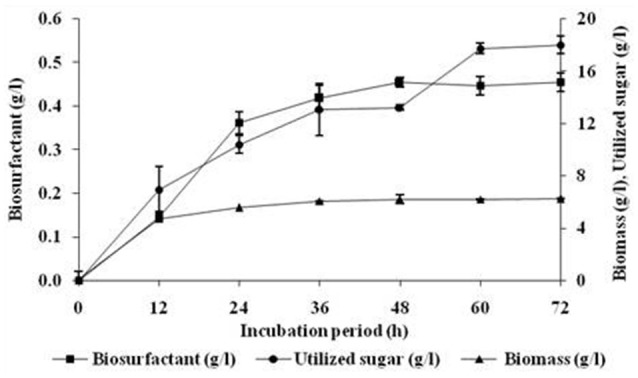
**Effect of incubation period on growth and biosurfactant production by ***B. subtilis*** ANR 88**. The isolate was inoculated at 2% concentration in minimal medium containing molasses (4%), ammonium ferric citrate (0.25%) as the nitrogen source and pH 7 incubated at 30°C at 160 rpm. Growth expressed in terms of biomass and biosurfactant extracted and estimated gravimetrically. Results are expressed as the average ± SD of three independent measurements.

As can be seen from Figure 1, the biosurfactant production started in the early growth phase and continued during the stationary phase. However, it is interesting to note that about 75% (0.362 g/l) of the biosurfactant was produced during the first 24 h and further production slowly reaching to final yield of 0.455 g/l after 48 h. The growth of the culture ceased at around 12 h but the utilization of sugar and production of biosurfactant continued further with biomass production reaching the final level after 36 h of incubation. The biosurfactant production by *B. subtilis* ANR 88 did not appear to be growth-associated.

### Medium optimization by Plackett-Burman design

The levels of media constituents optimized by OVAT method were used for deciding the range of levels of the constituents for Plackett-Burman optimization studies. Table 1 in materials and methods, shows the variables and their respective high or low levels selected for the optimization studies. Table 4 represents the PBD using the selected 11 variables for 12 runs with two levels of concentration. Table 4 includes the responses obtained in terms of biosurfactant yield (g/l) for each run. Medium components, *viz*., molasses, ammonium ferric citrate, magnesium sulfate, calcium chloride, manganese sulfate, ferrous sulfate, disodium phosphate, potassium di-hydrogen phosphate; culture conditions, such as, pH, size of the inoculum, and period of incubation were included as the variables in the PBD experiment.

**Table 4:** **PBD experimental design for 11 variables and the corresponding response values**.

**Runs**	**A**	**B**	**C**	**D**	**E**	**F**	**F**	**H**	**J**	**K**	**L**	**Biosurfactant yield (g/l)**	Surface tension^*^ (mN/m)
1	–	–	–	+	–	+	+	–	+	+	+	0.328 ± 0.014	27.305 ± 0.131
2	–	+	–	+	+	–	+	+	+	–	–	0.216 ± 0.025	28.329 ± 0.037
3	+	**–**	**–**	**–**	+	**–**	+	+	**–**	+	+	**0.746 ± 0.014**	27.923 ± 0.026
4	–	–	–	–	–	–	–	–	–	–	–	0.292 ± 0.024	25.833 ± 0.001
5	+	+	+	–	–	–	+	–	+	+	–	0.389 ± 0.081	27.425 ± 0.062
6	+	+	–	+	+	+	–	–	–	+	–	0.371 ± 0.050	26.729 ± 0.048
7	–	–	+	–	+	+	–	+	+	+	–	0.228 ± 0.026	29.234 ± 0.329
8	+	–	+	+	+	–	–	–	+	–	+	0.430 ± 0.008	26.700 ± 0.170
9	–	+	+	+	–	–	–	+	–	+	+	0.438 ± 0.012	28.380 ± 0.465
10	+	–	+	+	–	+	+	+	–	–	–	0.576 ± 0.107	27.442 ± 0.011
11	–	+	+	–	+	+	+	–	–	–	+	0.336 ± 0.064	26.441 ± 0.112
12	+	+	–	–	–	+	–	+	+	–	+	0.562 ± 0.076	28.005 ± 0.058

*+, High concentration levels; –, Low concentration levels*.

*Results are expressed as the average ± SD of three independent measurements*.

* *Surface Tension of the control: 50.001 ± 2.911 mN/m*.

*The optimal parameters and values are shown in bold*.

#### Significant variables

Table 4 shows the obtained response values in terms of biosurfactant yield (g/l) and surface tension measurements (mN/m) for the 12 experiments with the standard deviation. It may be seen that run 3 with 5% molasses as carbon source gave the highest response value of 0.746 g/l which is a remarkable 3.6-fold increase as compared to unoptimized yield of 0.207 g/l obtained from minimal medium with glucose. The response values (*Y*) were fitted to the best-fit linear equation and was obtained as follows:

In terms of actual factors,

(2) $$\begin{array}{r} Y=-\;0.33898\;+\;0.10321\times A\;+\;0.051625\times H-0.025229\\ \times \;J+\;0.064042\times L\ldots \end{array}$$

**Figure 2:**
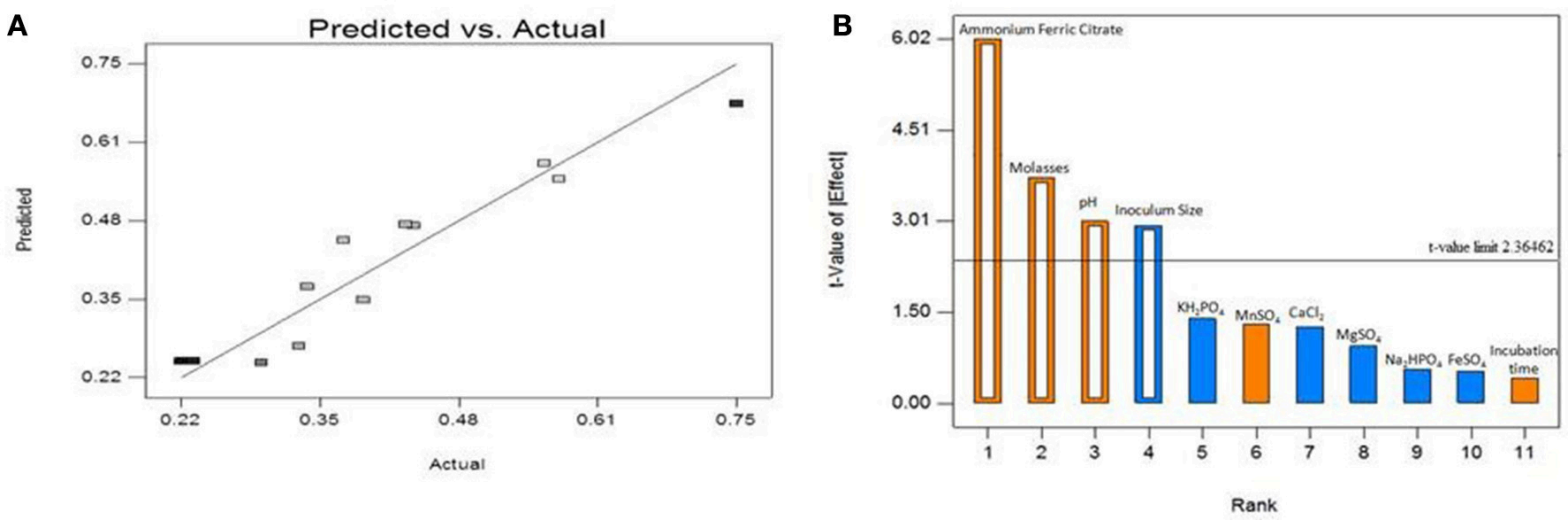
**(A)** Plot of model predicted vs. actual biosurfactant yield obtained from *B. subtilis* ANR 88 by PBD. **(B)** Pareto chart identifying the significant variables as those lying above the *t*-value limit.

The above equation shows that ammonium ferric citrate (*A)*, pH (*H*) and molasses (*L*) has significant positive effect while inoculum size (*J*) showed a negative effect on biosurfactant yield. The obtained model *F*-value of 16.98 implies that the model is significant and that there is only a 0.1% chance that at model *F-*value this large could occur due to noise. Values of “Prob > *F*” < 0.05 were obtained for all the **four** significant model terms forming the above equation with *R*^2^ = 0.9066. The predicted *R*^2^ of 0.7254 also lies in the range of adjusted *R*^2^ = 0.8532. The adequate precision measures the signal-to-noise ratio (SNR) and the obtained value of 11.418 indicates an adequate signal. Figure 2A represents the relationship between the actual biosurfactant concentration values and the predicted values determined by the model equation (2) with *B. subtilis* ANR 88. The points lie nearby the diagonal line with an equal distribution of all the points on both sides suggesting that the model explains the experimentally determined observations. The model can therefore be used to navigate the design space.

Table 4 indicates, that four out of eleven variables, *viz*. ammonium ferric citrate, molasses, pH and inoculum size had a significant influence on biosurfactant production. Their statistical significance is expressed in terms of % contribution in the Table 5. Figure 2B shows the Pareto chart of the effects displaying the relative levels of significance. Ammonium ferric citrate, the nitrogen source in the medium, showed the highest level of significance with a contribution of 48.37% with an *F*-value of 36.24 and a very low *p* < 0.0005. Thus, there is a significant positive effect between ammonium ferric citrate concentration and biosurfactant production. Molasses, the source of carbon in the medium also shows its significance in the production process with an 18.62% contribution, an *F*-value of 13.95 and *p* < 0.05. pH of the medium also showed positive correlation with the biosurfactant production in this study with 12.1% contribution, indicating the ability of the isolate to produce biosurfactant at alkaline pH. The *F*- and *p*-value were found to be 9.07 and 0.0196 respectively. Interestingly, inoculum size was found to have negative effect on biosurfactant production with a contribution of 11.56% and *F-* and *p*-values 8.66 and 0.0216 respectively.

**Table 5:** **Statistical parameters for the various components included in the PBD screening study**.

**Variables code**	**Media constituents**	**Effect**	**SS^a^**	**Contribution (%)**	***F*-value**	***p*-value^b^**	**Significance**
A	Ammonium Ferric Citrate (g/l)	0.21	0.13	48.37	36.24	0.0005	Yes
B	KH_2_PO_4_ (g/l)	−0.048	0.007	2.62			No
C	Na_2_HPO_4_ (g/l)	−0.02	0.001	0.435			No
D	MgSO_4_.7H_2_O (g/l)	−0.033	0.003	1.21			No
E	CaCl_2_ (g/l)	−0.043	0.0056	2.14			No
F	FeSO_4_.7H_2_O (g/l)	−0.018	0.001	0.38			No
G	MnSO_4_.4H_2_O (g/l)	0.045	0.006	2.32			No
H	pH	0.1	0.32	12.1	9.07	0.0196	Yes
J	Inoculum size (%)	−0.1	0.31	11.56	8.66	0.0216	Yes
K	Incubation Period (h)	0.014	0.001	0.24			No
L	Molasses (%)	0.13	0.049	18.62	13.95	0.0073	Yes

a *Sum of Squares*.

b *p < 0.05 were considered significant*.

#### Non-significant variables

The analysis shows that the identified non-significant variables, *viz*., ferrous sulfate, potassium di-hydrogen phosphate, di-sodium phosphate, magnesium sulfate and calcium chloride have negative effect on the biosurfactant production (Table 5). The buffering components, potassium di-hydrogen phosphate and di-sodium phosphate have no significant role in biosurfactant production but show negative effect of −0.048 and −0.02 respectively with 2.62 and 0.435% contribution, respectively. Magnesium sulfate, showed non-significant negative effect (−0.033) with a 1.21% contribution in the biosurfactant production by the isolate. Similarly, calcium chloride was seen to be a non-significant factor in the experiment indicating its requirement lies in the low level of the chosen experimental limits. It showed a negative effect of 0.043 with 2.14% contribution in the biosurfactant production. Ferrous sulfate exhibited negative effect of 0.018 and 0.38% contribution in the biosurfactant production process. The study showed that manganese sulfate showed non-significant but positive effect (0.045) with a 2.32% contribution on biosurfactant production. Incubation period was also identified as having the non-significant effect (0.014) with a 0.24% contribution in the production process.

### Characterization of biosurfactant by FT-IR

**Figure 3:**
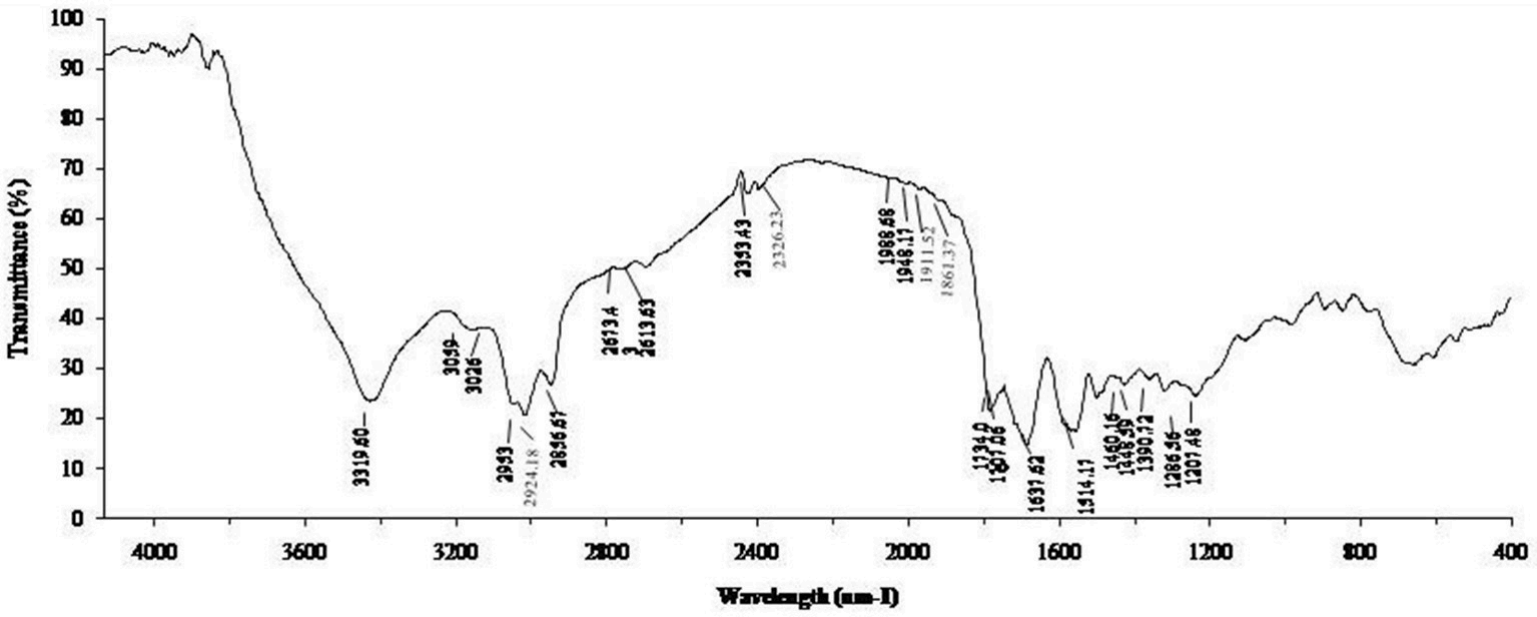
**FT-IR spectum of biosurfactant from ***B. subtilis*** ANR 88**.

The biosurfactant from *B. subtilis* ANR88 was characterized by Fourier Transform Infrared Spectroscopy to find out the nature of the biosurfactant produced (Figure 3). The characteristic absorbance bands of peptides at 3,319, 1,637, and 1,514 cm^−1^ (NH-stretching mode, CO-N bond and deformation mode of the N-H bond combined with C-N stretching) were observed. The absorbance peak at 2,953, 2,924, and 2,856 cm^−1^ and 1,460–1,390 cm^−1^ indicate the presence of alkyl chains (–CH2– and –CH3). The bands at 1,707 and 1,734 cm^−1^ were due to lactone carbonyl (C = O) absorption, corresponding to an ester carbonyl group. A weak absorbance signal at 1,448, 1,460 cm^−1^ is due to bending vibrations of C-H bonds associated with alkyl chains. Another absorption peak at 1,207 cm^−1^ is due to C-O stretching vibrations related to esters.

### Production of silver and gold nanoparticles from biosurfactant

Aqueous solution of biosurfactant extracted and purified as described earlier was incubated with AgNO_3_ or HAuCl_4_ for nanoparticle synthesis. Visual observation showed a color change from colorless to reddish brown and pinkish purple, clearly indicating the formation of SNP and GNP respectively. UV-Vis spectra of the mixture resulted in a single and strong peak at 420 nm and 540 nm indicating the formation of silver and gold nanoparticles respectively.

#### Silver nanoparticles synthesis

**Figure 4:**
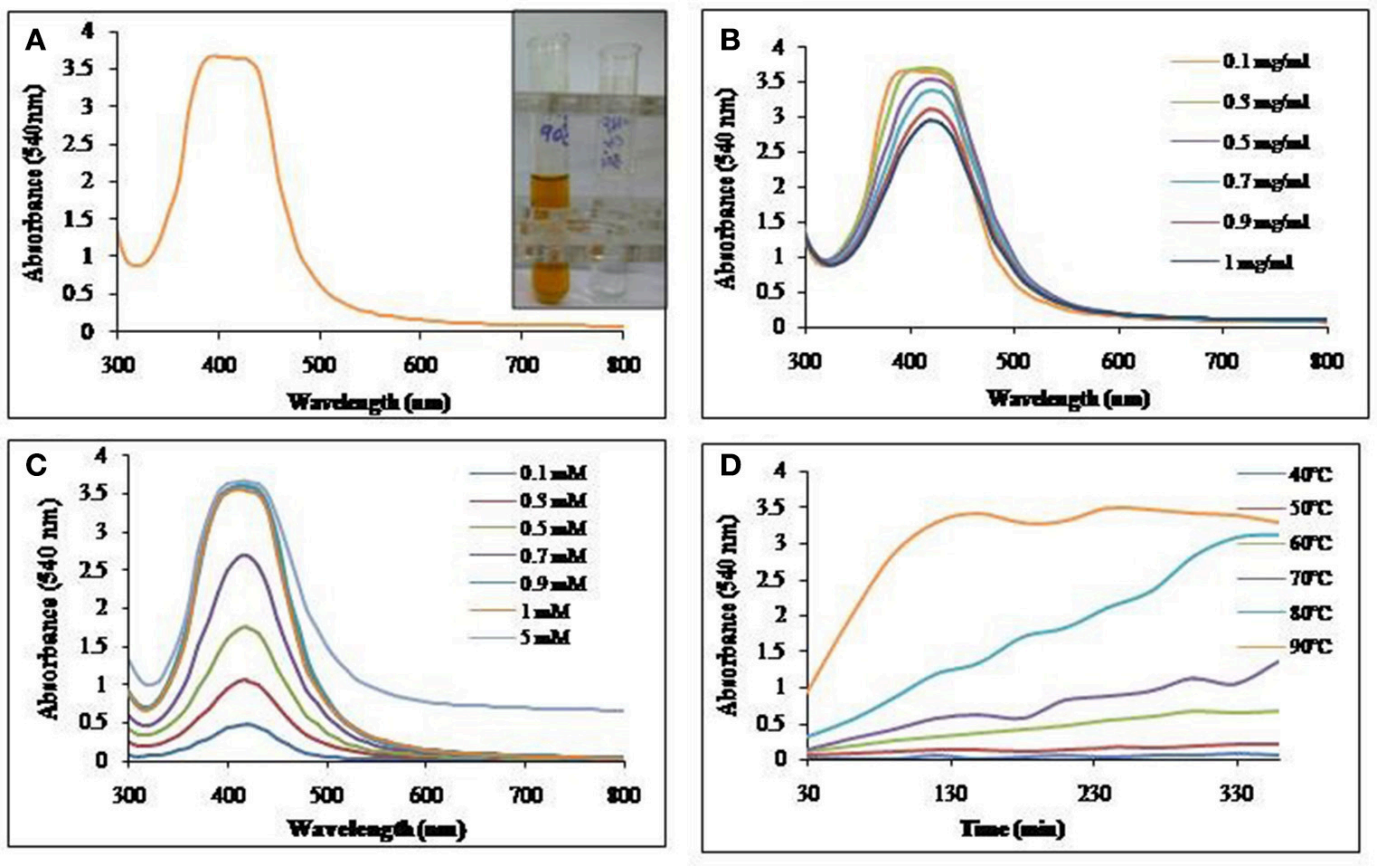
**UV-Vis absorption spectra of (A)** SNP synthesized using biosurfactant at 90°C. Inset: Visual color change due to SNP synthesis in the mixture of biosurfactant and AgNO_3_. **(B)** Effect of biosurfactant concentrations on SNP synthesis at 90°C. **(C)** Effect of AgNO_3_ concentration on SNP synthesis at 90°C and **(D)** Time course of synthesis of SNP at different temperatures.

Biosurfactant solution was incubated with the AgNO_3_ solution and as indicated in Figure 4A, SNP synthesis with visual color change, monitored with UV-Vis spectrophotometer, was observed with absorption maxima at 420 nm. The synthesis of SNP did not increase with the increasing concentration of biosurfactant (Figure 4B); however, increasing concentrations of AgNO_3_ resulted in increased SNP synthesis (Figure 4C). The absorbance at 420 nm was as low as 0.4 at 0.1 mM AgNO_3_ concentration, while it became 2.7 at 0.7 mM, AgNO_3_. An absorbance of 3.6 was seen at AgNO_3_ concentrations 0.9 mM and above. The biosynthesis of SNPs was carried out at different temperatures from 40 to 90°C (Figure 4D). Absorbance due to SNPs formed was found to increase at much higher rate at 90°C, while at 40 and 50°C there was no synthesis. As the temperature of reaction mixture was increased from 50 to 90°C, the rate of synthesis of SNP (in terms of absorbance at 420 nm) was found to increase.

**Figure 5:**
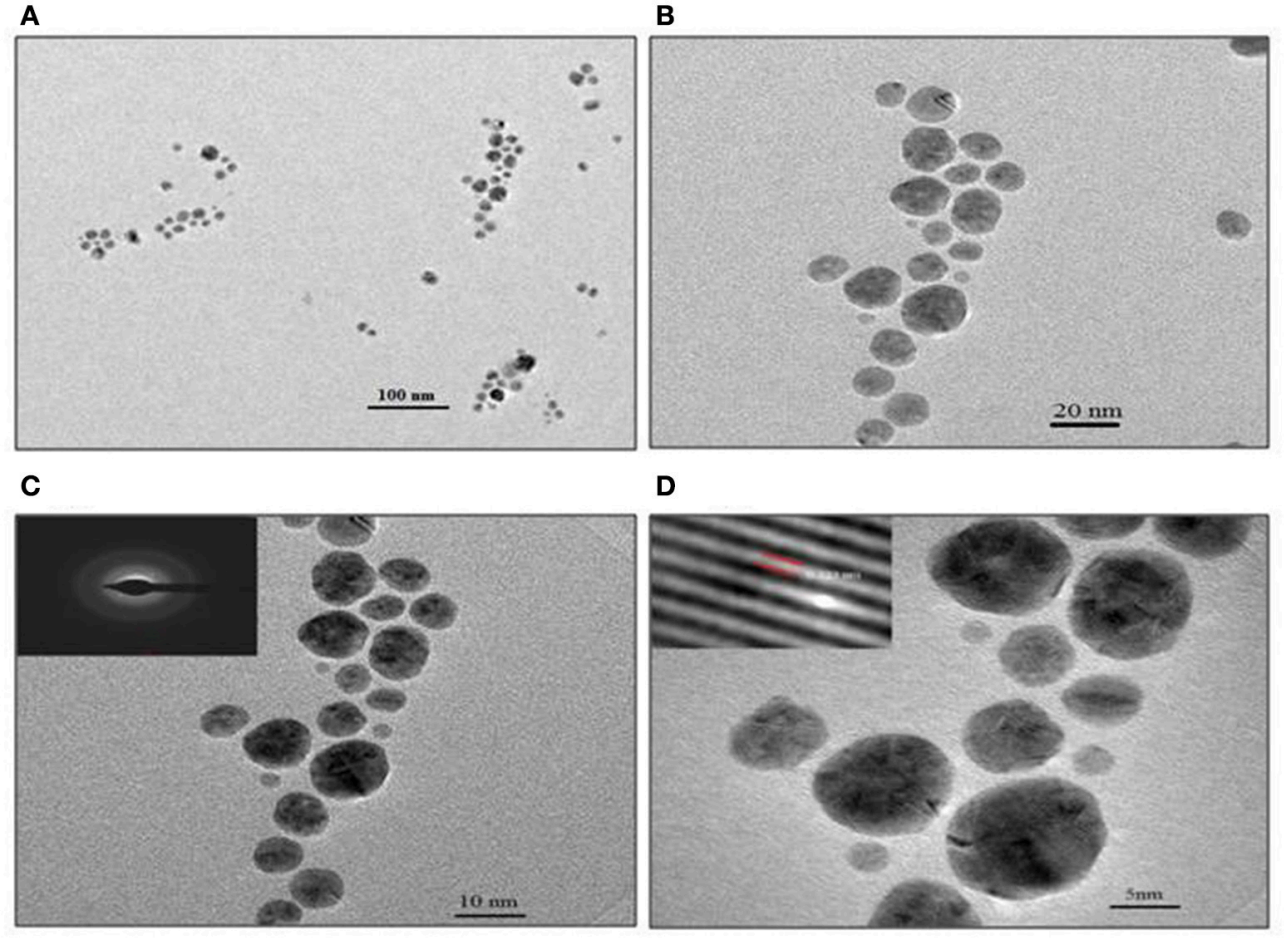
**TEM images of SNP synthesized with 0.1 mg/ml biosurfactant and 0.9 mM AgNO_**3**_, incubated at 90°C**. Images of nanoparticles at various resolutions are shown: **(A)** 100 nm, **(B)** 20 nm, **(C)** 10 nm Inset: SAED pattern and **(D)** 5 nm scale. Inset: Lattice fringes of SNP.

TEM analysis revealed predominantly spherical nature of particles and in the size range of 4–18 nm (Figure 5). Figures 5A,B show the SNPs at 100 nm and 20 nm resolution. The selected area electron diffraction pattern (SAED) in Figure 5C shows concentric rings with intermittent bright dots, indicating that these nanoparticles were crystalline in nature. Distinct lattice fringes were seen in the images with higher magnification (Figure 5D), wherein, the distance between two successive lattice fringes was 0.223 nm, typically observed for the crystalline structure of the SNP (Singh et al., 2013).

#### Gold nanoparticle synthesis

**Figure 6:**
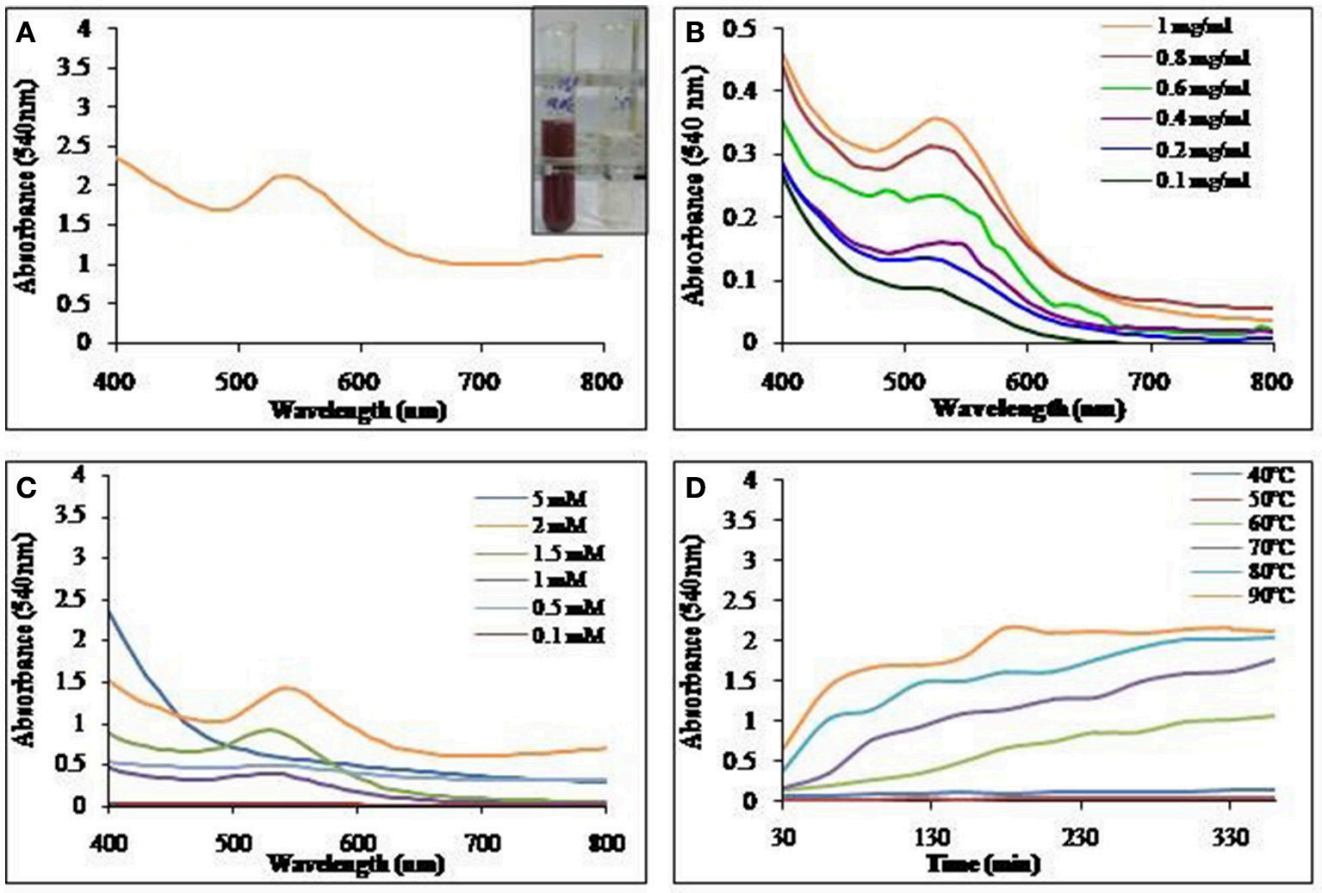
**UV-Vis absorption spectra of (A)** GNP synthesized using biosurfactant at 90°C. Inset: Visual color change due to GNP synthesis in the mixture of biosurfactant and HAuCl_4_. **(B)** Effect of biosurfactant concentration on GNP synthesis at 90°C. **(C)** Effect of HAuCl_4_ concentration on GNP synthesis at 90°C and **(D)** Time course of synthesis of GNP at different temperatures.

Biosurfactant solution incubated with HAuCl_4_, yielded GNPs with visual color change as seen in Figure 6A. The GNP synthesis was confirmed by UV-Vis spectrum of the colored solution, with absorption maxima at 540 nm. Synthesis of GNP increased with increase in biosurfactant concentration. Maximum GNP synthesis was observed at 1 mg/ml (Figure 6B). GNP synthesis also increased with increase in HAuCl_4_ concentration till 2 mM, above which, there was no synthesis (Figure 6C). It was found that there was an increase in the rate of synthesis of GNP with rise in temperature of incubation (Figure 6D).

**Figure 7:**
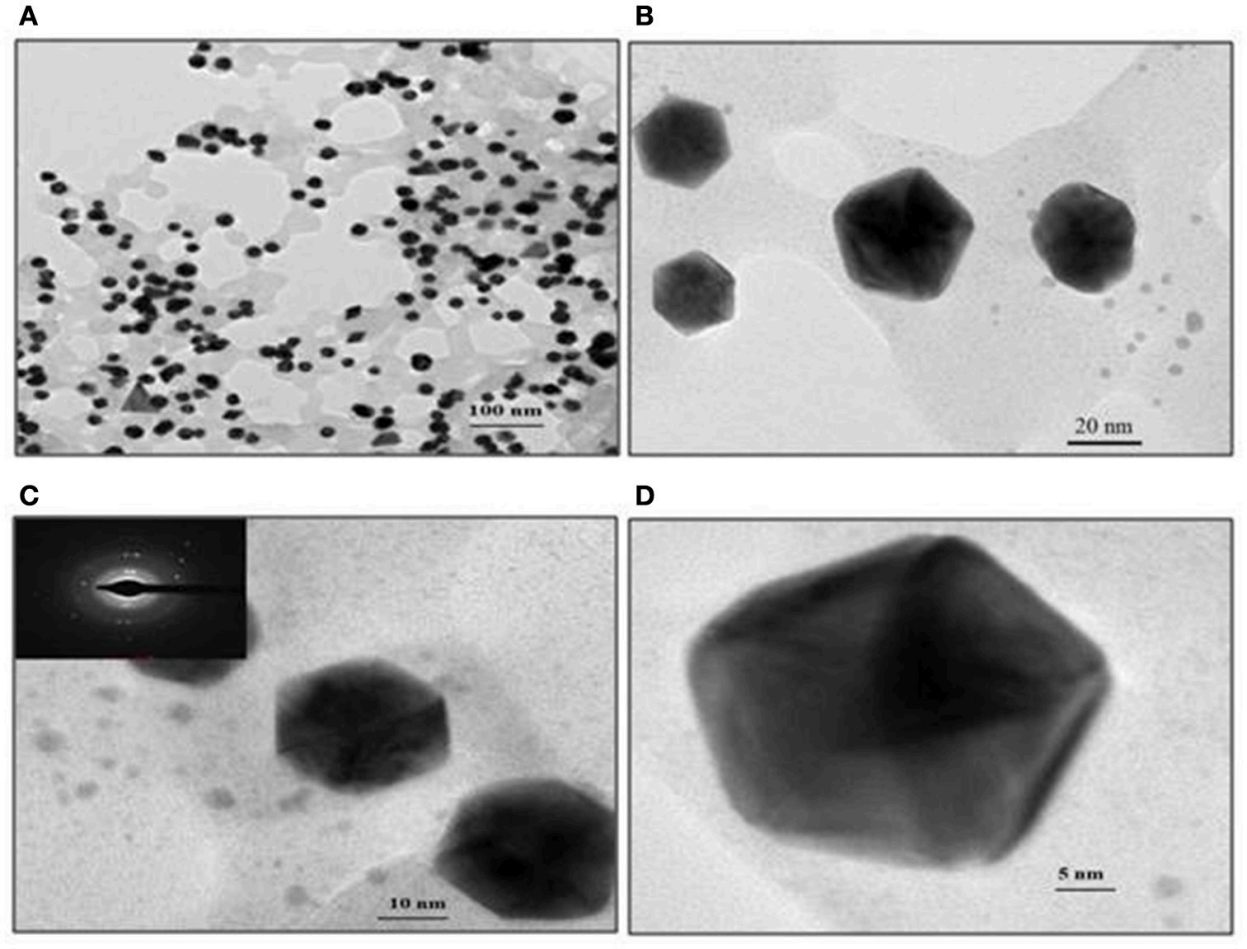
**TEM images of gold nanoparticles synthesized with 1 mg/ml biosurfactant and 2 mM HAuCl_**4**_, incubated at 90°C**. Images of nanoparticles at various resolutions are shown: **(A)** 100 nm, **(B)** 20 nm, **(C)** 10 nm and **(D)** 5 nm scale. Inset in **(C)**: Corresponding SAED pattern

Representative TEM images of the GNP, depicted in Figure 7, show the biosurfactant mediated GNP synthesis with varying size and shapes. Spherical, triangular and mostly hexagonal nanoparticles were observed varying in size between 40 and 60 nm. SAED profile of the GNPs synthesized is shown in inset of Figure 7C. This profile of concentric rings with intermittent bright spots confirmed the crystalline nature of the nanoparticles. Representative TEM images of the GNP, depicted in Figure 7, show the biosurfactant mediated GNP synthesis varying in the size and shapes. Spherical, triangular and mostly hexagonal nanoparticles were observed with size lying between 40 and 60 nm. SAED profile of the GNPs synthesized is shown in the inset of Figure 7C. The profile of concentric rings with intermittent bright spots confirmed the crystalline nature of the nanoparticles.

## Discussion

Biosurfactants are microbial products having the amphiphilic structure, where lipid component confers hydrophobicity, while carbohydrates, peptides and phosphates confer hydrophilic properties. The amphiphilic nature is responsible for use of biosurfactant for formation of emulsions (*viz*., water in hydrocarbon or hydrocarbon in water) which lead to solubilization of hydrocarbons in polar aqueous solvents. Structure, function, and properties of biosurfactants have been extensively reviewed (Banat et al., 2010). Because of the biodegradable nature of biosurfactants, they are more environmental friendly than chemical surfactants. Biosurfactants have wide array of applications in oil industry, especially for oil recovery and pollution control. Also, potential applications of biosurfactant in agriculture, bioremediation, petrochemical, pharmaceutical, cosmetics, detergent and food industries have been well recognized (Gudiña et al., 2015). Although use of biosurfactants has important advantages as discussed earlier, its high cost of production severely limits their extensive use when compared to synthetic chemical surfactants. Improving the productivity as shown here is a step toward making the use of biosurfactants affordable.

Majority of known biosurfactants are obtained from microbial cultures grown on hydrocarbons or other water immiscible substrates. There are few reports of production of biosurfactants with water soluble growth substrates (Bordoloi and Konwar, 2008). In the present work, the production of biosurfactant was carried out using water soluble/miscible, cheap agro-industrial wastes as growth substrates and indigenously isolated *B. subtilis* ANR 88. Production of biosurfactant by the isolate on molasses medium was similar to that on glucose as carbon source. The ability of the culture to produce biosurfactant using molasses in place of sucrose and/or glucose would prove significant in reducing the cost of production. Interestingly, though the yield of biosurfactant was less with potato peels extract, the specific yield of biosurfactant per gram of utilized sugar was maximum as compared to that on glucose and molasses. The very low nutritional quality of potato peels extract may be forcing the culture to produce biosurfactant in an attempt to survive. It is interesting to point out that the medium with potato peels extract contained the minimum sugar concentration.

The production of biosurfactant by *B. subtilis* ANR 88 was optimized using OVAT and PBD approach, resulting into yields of 0.745 g/l, which is about 3.6 times than that with naive medium with glucose or molasses as carbon source. The production of biosurfactant was concentration dependent with molasses as carbon source. Joshi et al. (2008a), in their studies on *B. subtilis* 20B and *Bacillus* strain HS3 have reported inhibitory effect of molasses (more than 9%). However, biosurfactant production by our isolate, *B. subtilis* ANR 88 was not affected by 10% molasses, as seen in the present study.

Amongst inorganic nitrogen sources, (*viz*., ammonium nitrate, ammonium ferric citrate, ammonium sulfate, ammonium chloride, potassium nitrate, and sodium nitrate), ammonium ferric citrate was found to be the most suitable nitrogen source for biosurfactant production by *B. subtilis* ANR 88. The necessity of iron for biosurfactant production by *Pseudomonas aeruginosa* strain was shown by Gudiña et al. (2015). The isolate *B. subtilis* ANR 88 produced biosurfactant in the wide range of pH (5–9), so also the temperature range for production was from 25 to 37°C.

Results of FT-IR strongly indicate that the biosurfactant contained aliphatic and peptide-like moieties (De Faria et al., 2011; Pereira et al., 2013; Varadavenkatesan et al., 2013). The absorbance peaks at 3,319 cm^−1^ along with that at 1,637 cm^−1^ and 1,514 cm^−1^ in FT-IR analysis is strong indication of presence of peptide bonds; these absorbance peaks are resulting from NH-stretching mode, CO-N bond and deformation mode of the N-H bond combined with C-N stretching. Presence of alkyl chains (absorbance bands at 2,953, 2,924, and 2,856 cm^−1^ and 1,460–1,390 cm^−1^) along with C-N bonds confirm the lipopeptide nature of the biosurfactant (Joshi et al., 2008b).

There is considerable interest in biosurfactant mediated nanoparticles synthesis as it is thought to be environmentally benign process (Plaza et al., 2014). Mukherjee et al. (2002) and Ahmad et al. (2003) have reported the biological synthesis of gold and silver nanoparticles using microorganisms intracellularly and extracellularly. Reddy et al. (2009a,b) used NaBH_4_ as a reducing agent in the synthesis of SNPs and GNPs using foam fractionated biosurfactant as the stabilizer. Kiran et al. (2010) and Kumar C. G. et al. (2010) have shown independently the use of glycolipids and rhamnolipids respectively in nanoparticles stabilization. Kasture et al. (2008) and Kumar D. R. et al. (2010) have suggested the role of sophorolipids biosurfactant as reducing and capping agents in nanoparticles synthesis process. In present study, we obtained the SNP and GNP synthesis using lipopeptide biosurfactant from *B. subtilis* ANR 88 in the absence of any chemical reducing agent. We therefore feel that the biosurfactant of *B. subtilis* ANR 88 is capable of acting as both reducing as well as stabilizing agent in silver and gold nanoparticle synthesis.

## Conclusion

In this study, we have utilized cheap, unconventional agro-industrial wastes, *viz*., molasses, orange peels extract, bagasse extract, banana peels extract and potato peels extract to produce biosurfactant from the indigenously isolated *Bacillus subtilis* ANR 88 with significant advantages realized for their application in nanoparticle synthesis. Silver and gold nanoparticle synthesis with biosurfactant from *B. subtilis* ANR 88 did not require chemical reducing agent, making it a green alternative to its chemical counterparts.

## Author contributions

AR has designed and carried out the research work and wrote the manuscript. VB has contributed in terms of valuable assistance in the execution of part of the experimental work and in the preparation of manuscript. VR designed and analyzed the Plackett-Burman experimental design and provided valuable suggestions in manuscript preparation. RD formulated the entire research, supervised it and contributed in the manuscript preparation.

### Conflict of interest statement

The authors declare that the research was conducted in the absence of any commercial or financial relationships that could be construed as a potential conflict of interest.
