# Corrigendum: Agro-Industrial Wastes for Production of Biosurfactant by *Bacillus subtilis* ANR 88 and Its Application in Synthesis of Silver and Gold Nanoparticles

**DOI:** 10.3389/fmicb.2017.00878

**Published:** 2017-05-16

**Authors:** Ashwini N. Rane, Vishakha V. Baikar, V. Ravi Kumar, Rajendra L. Deopurkar

**Affiliations:** ^1^Department of Microbiology, Savitribai Phule Pune UniversityPune, India; ^2^Department of Environmental Science, Savitribai Phule Pune UniversityPune, India; ^3^Chemical Engineering and Process Development Division, CSIR-National Chemical LaboratoryPune, India

**Keywords:** biosurfactant, agro-industrial waste, nanoparticles, *Bacillus subtilis*, Plackett-Burman design-optimization

An author name was incorrectly spelled as D. V. Ravi Kumar. The correct spelling is V. Ravi Kumar. The authors apologize for this error and state that this does not change the scientific conclusions of the article in any way.

The Original Article has been updated.

## Conflict of interest statement

The authors declare that the research was conducted in the absence of any commercial or financial relationships that could be construed as a potential conflict of interest.

